# Pulsatility of insulin release – a clinically important phenomenon

**DOI:** 10.3109/03009730903366075

**Published:** 2009-12-08

**Authors:** Bo Hellman

**Affiliations:** Department of Medical Cell Biology, Uppsala University, UppsalaSweden

**Keywords:** ATP, calcium oscillations, diabetes, glucagon, insulin, islets of Langerhans, purinergic receptors, somatostatin

## Abstract

The mechanisms and clinical importance of pulsatile insulin release are presented against the background of more than half a century of companionship with the islets of Langerhans. The insulin-secreting β-cells are oscillators with intrinsic variations of cytoplasmic ATP and Ca^2+^. Within the islets the β-cells are mutually entrained into a common rhythm by gap junctions and diffusible factors (ATP). Synchronization of the different islets in the pancreas is supposed to be due to adjustment of the oscillations to the same phase by neural output of acetylcholine and ATP. Studies of hormone secretion from the perfused pancreas of rats and mice revealed that glucose induces pulses of glucagon anti-synchronous with pulses of insulin and somatostatin. The anti-synchrony may result from a paracrine action of somatostatin on the glucagon-producing α-cells. Purinoceptors have a key function for pulsatile release of islet hormones. It was possible to remove the glucagon and somatostatin pulses with maintenance of those of insulin with an inhibitor of the P2Y_1_ receptors. Knock-out of the adenosine A_1_ receptor prolonged the pulses of glucagon and somatostatin without affecting the duration of the insulin pulses. Studies of isolated human islets indicate similar relations between pulses of insulin, glucagon, and somatostatin as found during perfusion of the rodent pancreas. The observation of reversed cycles of insulin and glucagon adds to the understanding how the islets regulate hepatic glucose production. Current protocols for pulsatile intravenous infusion therapy (PIVIT) should be modified to mimic the anti-synchrony between insulin and glucagon normally seen in the portal blood.

## Introduction

For more than half a century pancreatic islets have been intensely studied at the University of Uppsala. After examining the mechanisms for alloxan destruction of the insulin-producing β-cells (1) and the principles for dissemination of the endocrine pancreas into islets (2), most of my attention has been paid to the insulin secretory process. Early studies of isolated islets made it possible to propose that glucose stimulation of insulin release is mediated by increase of the cytoplasmic Ca^2+^ concentration ([Ca^2+^]_i_) in pancreatic β-cells (3). This idea was confirmed by direct measurements of [Ca^2+^]_i_ (4). Even more important, use of ratiometric fura-2 technique demonstrated the existence of oscillatory rises of [Ca^2+^]_i_ (5,6) that triggered 3–4 min pulses of insulin release (7).

A prerequisite for pulsatile release of islet hormones is that the [Ca^2+^]_i_ oscillations are entrained into a common rhythm in the cells involved. Accumulating data indicate that both individual cells and whole islets behave as coupled oscillators (8,9). Like other limit-cycle oscillators the β-cells are expected to synchronize when the coupling signal is sufficient to overcome the differences in natural frequencies. We imagine that the synchronization emerges co-operatively, analogous to phase transitions such as freezing of water or spontaneous magnetization of a ferromagnet (10,11). In accordance with phase transitions, the alignment of islet cell oscillations in time may be the counterpart of the alignment of molecules in space.

This review presents the author's views about the mechanism and clinical importance of pulsatile insulin release with emphasis on recent contributions from our laboratory. Besides examining how β-cells generate pulses of insulin release it is discussed how these cells co-ordinate their rhythmicity. The finding that pulses of insulin are anti-synchronous to those of glucagon adds to the understanding of the islet regulation of hepatic glucose production.

## Insulin pulses are triggered by intrinsic β-cell rhythmicity of ATP and Ca^2+^

A number of studies have indicated periodic variations of circulating insulin in the peripheral blood (12,13). Initially, these oscillations were supposed to reflect pulsatile release of insulin generated by the central nervous system. This idea was refuted after the observation that insulin is released from the isolated pancreas in a pulsatile fashion (14). The controversies about the pacemaker for pulsatile insulin release were settled after our observation that β-cells have the intrinsic ability to generate 2–5-min [Ca^2+^]_i_ oscillations resulting from periodic depolarization (5,6).

In 1969 we organized an international symposium as a centennial of Paul Langerhans' discovery of the islets (15). At that time it was generally accepted that glucose depolarizes the β-cells via its metabolism. A few years later colleagues in Umeå provided the first evidence that the glucose-induced depolarization of the β-cells was mediated by suppression of the K^+^ permeability (16). The introduction of the patch clamp technique (17) made it possible to demonstrate that β-cells have K^+^ channels inhibited by cytoplasmic ATP. The discovery of the K_ATP_ channel was made in Oxford (18), but many of its properties were first described in a thesis from Uppsala (19).

**Figure 1.**
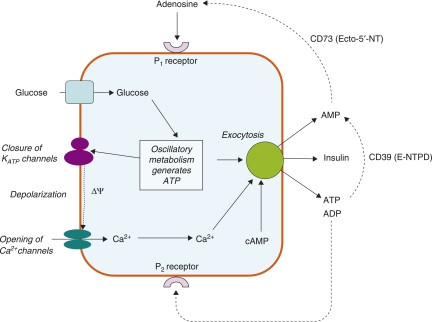
Model showing how glucose-induced oscillations of cytoplasmic ATP generate pulsatile release of insulin from a β-cell. Rhythmic glycolysis triggers periodic rises of cytoplasmic ATP that inhibits a specific K^+^ channel. Resulting depolarization evokes oscillations of cytoplasmic Ca^2+^ due to entry of the ion via voltage-dependent channels. The right part of the Figure shows that oscillatory rises of cytoplasmic Ca^2+^, ATP, and cAMP evoke exocytosis of secretory granules containing insulin together with ATP, ADP, and AMP. After release from the β-cell these nucleotides, degraded or not to adenosine by ectonucleotidases (CD39 and CD73), serve as regulators of pulsatile release of insulin by binding to P1 and P2 receptors.

The key role of ATP for generation of insulin release pulses is schematically illustrated in Figure 1. The cytoplasmic concentration of ATP (20) as well as the ATP/ADP ratio (21) are known to oscillate in glucose-stimulated β-cells. The metabolism of glucose induces periodic rises of cytoplasmic ATP due to oscillatory glycolysis mediated by the allosteric enzyme phosphofructokinase-M (22). Increase of ATP promotes insulin release by closure of the K_ATP_ channels with subsequent depolarization and influx of Ca^2+^ via voltage-dependent channels. Moreover, ATP sensitizes the secretory machinery to the Ca^2+^ signal, an effect amplified by cyclic AMP derived from ATP. Studies in our laboratory have shown that glucose induces periodic variations of cyclic AMP and plasma membrane phosphoinositide lipids (23–25). Glucose generation of pronounced oscillations of phosphatidylinositol 3,4,5-trisphosphate has been attributed to periodic release of insulin stimulating its receptors on the surface of the β-cells (26).

Besides acting as a cytoplasmic initiator of pulsatile insulin release ATP is a component of the secretory granules (Figure 1). After periodic release via exocytosis, ATP serves as an autocrine and paracrine messenger by activating purinergic P2 receptors (27). The P2 receptors belong to two major families: the ionotropic ligand-gated ion channel P2X and the metabotropic G-protein-coupled P2Y. Studies in our laboratory have shown that P2 receptors are important regulators of pulsatile insulin release from the β-cells (9,28,29). One of several mechanisms is periodic activation of phospholipase A_2_ with generation of arachidonic acid, a substance known to inhibit the K_ATP_ channels (30). Extracellular ATP is degraded by ectonucleotidases to adenosine, which binds to P1 receptors. These receptors have modulatory effects on islet hormone release by affecting the amplitude and duration of the pulses (29,31).

## Individual islet cells generate spontaneous Ca^2+^ oscillations with different frequencies

**Figure 2.**
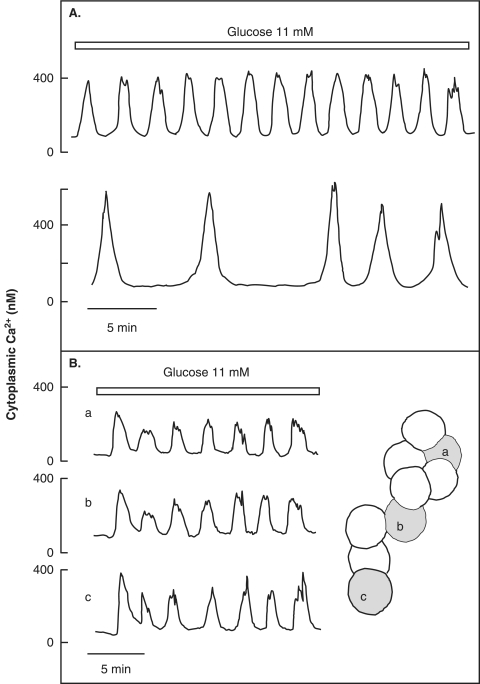
Oscillations of cytoplasmic Ca^2+^ in two mouse β-cells lacking contact (A) and in three cells situated in an aggregate (B). Contacts between the cells result in synchronization of the Ca^2+^ oscillations. The traces refer to the cells shown to the right.

It is difficult to study pulsatile release of insulin from individual β-cells without disturbing the spontaneous rhythm. The knowledge of the intrinsic rhythmicity of β-cells is therefore based essentially on measurements of [Ca^2+^]_i_. The observation that glucose generates oscillations of [Ca^2+^]_i_ in single β-cells was first reported from Uppsala (32). The oscillatory frequency differs considerably among β-cells lacking contact (Figure 2A). However, when situated in aggregates the β-cells are entrained into a common rhythm of about 0.3/min (Figure 2B). The oscillatory activity is critically dependent on sufficient oxygen supply during the isolation of the β-cells and procedures to minimize the exposure to UV light during the measurements of [Ca^2+^]_i_. Periodic rises of [Ca^2+^]_i_ were seen not only in β-cells but also in glucagon-producing α-cells (33), somatostatin-producing δ-cells (33), and pancreatic polypeptide-producing (PP) cells (34).

## β-Cells communicate via diffusible factors generating cytoplasmic Ca^2+^ transients

**Figure 3.**
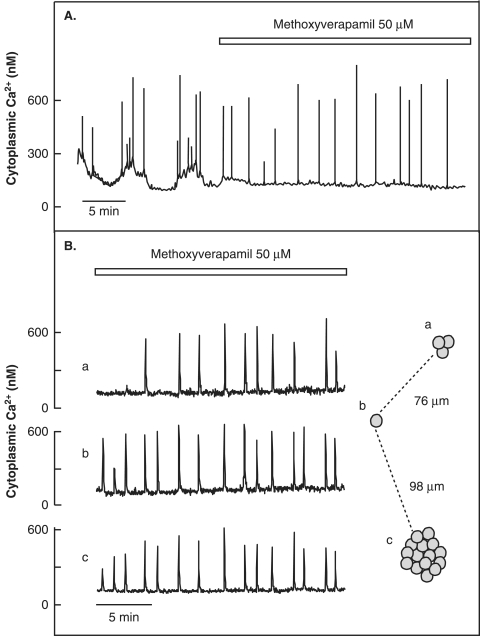
Transients of cytoplasmic Ca^2+^ in *ob/ob* mouse β-cells superfused with a medium containing 20 mM glucose and 20 nM glucagon. A: Suppression of the Ca^2+^ entry with methoxyverapamil removes the Ca^2+^ oscillations, allowing the transients to start from the basal level. B: Synchronized cytoplasmic Ca^2+^ transients in single cell/aggregates (shown to the right) exposed to methoxyverapamil.

β-Cell oscillations of [Ca^2+^]_i_ are sometimes superimposed with transients mediated by inositol 1, 4, 5 trisphosphate (IP_3_). Comparison of different animal models revealed that β-cells from obese-hyperglycaemic mice (*ob/ob*) are unusual by generating numerous [Ca^2+^]_i_ transients (35,36). Studies of these animals have been important for demonstrating that β-cells can communicate via diffusible factors. After eliminating the background of slow oscillations (inhibition of the Ca^2+^ entry), it was found that the transients propagate between adjacent β-cells lacking contact (Figure 3). In the attempts to identify the messengers involved, evidence was provided for regenerative release of ATP (37) and NO (38).

**Figure 4.**
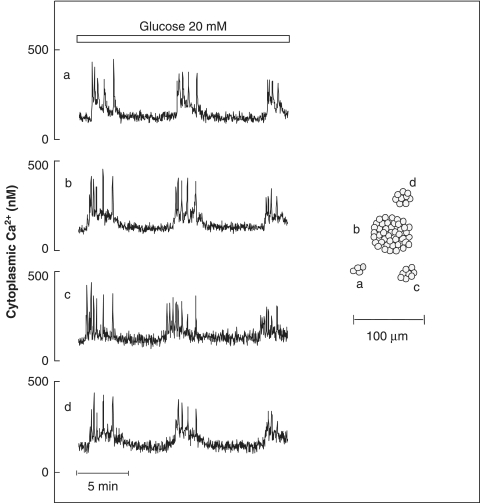
Co-ordination of [Ca^2+^]_i_ oscillations in the four aggregates shown to the right. Most of the superimposed transients appear in synchrony not only within but also among the aggregates. Modified from Grapengiesser et al. 2003 (8) with permission.

We have tested whether the transients of [Ca^2+^]_i_ have a co-ordinating action on the oscillatory activity in isolated β-cells (8). The experimental conditions were designed to promote IP_3_ generation of transients (*ob/ob* mouse β-cells exposed to 20 mM glucose and 20 nM glucagon). It was seen that β-cells/aggregates superimposed with synchronized transients are entrained into a common rhythm (Figure 4). The superimposed transients had a co-ordinating action on [Ca^2+^]_i_ oscillations in β-cells separated by a distance of < 100 μm, but not in those situated > 200 μm apart. There are other pathways for generating [Ca^2+^]_i_ transients than those mediated by IP_3_. Studies in our laboratory indicate prominent β-cell transients of [Ca^2+^]_i_ resulting from intermittent entry of the ion (39,40). Entry of Ca^2+^ via rapidly inactivating P2X receptors represents an attractive alternative for generation of the [Ca^2+^]_i_ rises supposed to entrain the glycolytic oscillator into a common rhythm.

## Synchronization of β-cells within and among the islets in rodents

Parallel measurements of [Ca^2+^]_i_ and release of insulin from single mouse islets support the idea that glucose-induced oscillations of [Ca^2+^]_i_ generate pulses of insulin (7). The role of glucose is both to induce [Ca^2+^]_i_ oscillations (41,42) and to make the exocytotic machinery more sensitive to the Ca^2+^ signal (43). Within the islets the β-cells are well co-ordinated, as indicated by the presence of synchronized [Ca^2+^]_i_ oscillations and distinct pulses of insulin release from the whole islet. There is a need for a strong coupling force to overcome the differences in the endogenous β-cell rhythm. A major part of this coupling is mediated by gap junctions made of connexin-36 (44,45). Regenerative release of ATP and London other messengers contribute to the co-ordination by propagating [Ca^2+^]_i_ transients between the β-cells. In very large islets the coupling mechanisms are insufficient to synchronize all β-cells, as reported from measurements of [Ca^2+^]_i_ in *ob/ob* mouse islets (46).

Each islet is an oscillatory unit, generating 3–4-min pulses of insulin release (47). Contrary to the co-ordination of β-cells within an islet, the synchronization of the islets in the pancreas requires a weak coupling force due to similarities in pulse frequency. The entrainment of the islets into the same oscillatory phase is very efficient, as indicated by the distinct pulses of hormone release from the perfused rat pancreas (48–50). The co-ordination of the β-cells from the different islets in the pancreas is supposed to be mediated by neural input from local ganglia (51,52). It was recently reported that repetitive pulses of the neurotransmitter acetylcholine, contrary to ATP, have a synchronizing action on isolated mouse islets (53,54). Mathematical modelling supported the idea that acetylcholine-induced increase of [Ca^2+^]_i_ resets the glycolytic oscillator. However, there are reasons to believe that the  failure to demonstrate a synchronizing effect of ATP was due to rapid desensitization of purinergic P2 receptors.

## Pulsatile release of islet hormones in rodents

**Figure 5.**
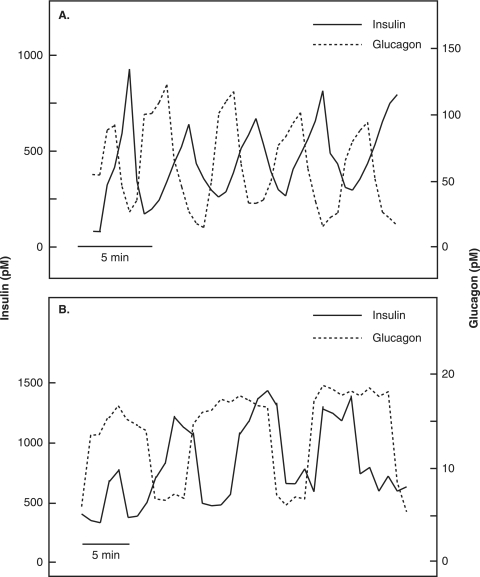
Relation between repetitive insulin and glucagon pulses during perfusion of rodent pancreas with 20 mM glucose. A: The pulses of insulin are anti-synchronous to those of glucagon in rat pancreas. From Grapengiesser et al. 2006 (49) with permission. B: The pulses of glucagon are prolonged compared with insulin in mice with knock-out of the adenosine A_1_ receptor. From Salehi et al. 2009 (31) with permission.

Like β-cells, the glucagon-producing α-cells and the somatostatin-producing δ-cells have an intrinsic ability to generate oscillations of [Ca^2+^]_i_. Entrained into a common rhythm these oscillations trigger pulses of hormone release. Increase of glucose from 3 to 20 mM generated pronounced pulses of insulin, glucagon, and somatostatin from the perfused pancreas of rats (48–50) and mice (31). The major component of hormone release was pulsatile, irrespective of whether the rhythmicity resulted in increase (insulin and somatostatin) or decrease (glucagon) of average secretory rate. Remarkably, the pulses of glucagon were anti-synchronous to those of insulin and somatostatin (49,50). The presence of reversed cycles of insulin and glucagon (Figure 5A) is much to the purpose, since these hormones have counteractive effects on the hepatic glucose production.

The mechanisms for regulation of pulsatile release of islet hormones are far from elucidated. Enhanced secretory rates of insulin (13,55) and glucagon (56) are usually related to increase of the pulse amplitude. Other studies have shown that glucose-induced oscillations of [Ca^2+^]_i_ are transformed into sustained elevation, when single mouse β-cells or intact islets are exposed to amino acids (57) or noxious agents (58). A pertinent question is whether the anti-synchrony between insulin and glucagon is removed by alterations of the pulse duration. The answer is yes (Figure 5B). Knock-out of the adenosine A_1_ receptor was found to prolong the pulses of glucagon and somatostatin but not of insulin (31). Interestingly, the pulsatile insulin release from rat pancreas persisted when the pulses of glucagon and somatostatin were suppressed by a low concentration of an inhibitor of the purinergic P2Y_1_ receptor (50).

**Figure 6.**
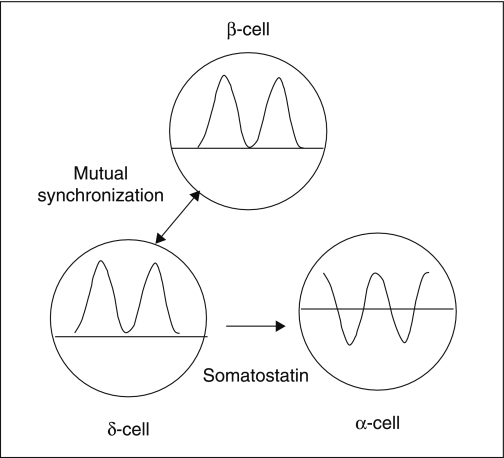
Model of cell interactions important for glucose generation of pulsatile hormone release from an islet. Dotted lines indicate level of basal release before the rise of glucose. Glucose generates simultaneous pulses of insulin and somatostatin release by mutual synchronization of β- and δ-cells. The oscillations of glucagon appear in anti-synchrony and have nadirs below the basal level. Paracrine release of somatostatin from δ-cells accounts for the appearance of glucagon pulses 180° out of phase.

The observation that islet hormones are released as pulses, with glucagon in anti-phase with insulin and somatostatin, makes it necessary to reconsider previous ideas how α-, β-, and δ-cells interact within rodent islets. A tentative model is presented in Figure 6. It is proposed that β- and δ-cells are entrained into a common rhythm due to mutual synchronization mediated by gap junctions and diffusible factors (ATP). The coupling between the two types of cells is weak, as suggested by the preservation of the insulin oscillations after prolongation (31) or removal (29,50) of the somatostatin pulses. Our model proposes that somatostatin, a well established inhibitor of secretion, modulates the pulsatile release of glucagon from the α-cells. The observation that periods with a rise of somatostatin are related to decrease of glucagon reinforces existing arguments that δ-cells have tonic inhibitory effects on α-cells (59–61). Such a paracrine action may well explain why glucagon pulses are in anti-phase with pulses of somatostatin and consequently also with insulin.

## Pulsatile release of islet hormones in man

**Figure 7.**
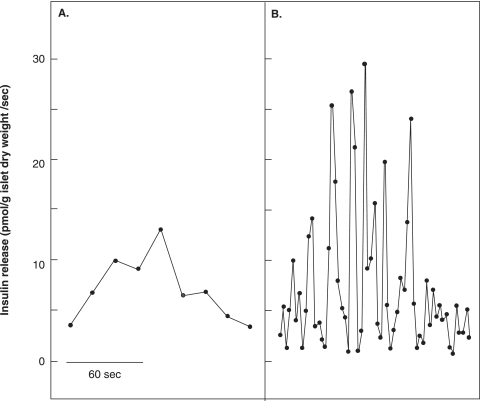
Insulin release from a human islet exposed to 11 mM glucose. A: Pulse observed with a sampling time of 17.5 seconds. B: The same pulse analysed with a sampling time of 2.5 seconds.

Privileged with access to human islets for more than 15 years we now conclude that the mechanisms for pulsatile release of islet hormones in man resemble those in rats and mice. It was possible to show that also human β-cells have specific K^+^ channels regulated by cytoplasmic ATP (28). The activity of these channels varied with the same periodicity as the depolarizing waves triggering the Ca^2+^ entry into the β-cells (62). The similarity with rodents refers not only to the [Ca^2+^]_i_ rises that trigger insulin pulses but also to kinetics of hormone release (28). Measurements of insulin release with high time resolution revealed that secretory pulses from single islets can be resolved into episodes of 10–20 seconds (Figure 7). These episodes reflect the bursts of electrical activity characteristic of β-cells situated in intact islets (63).

**Figure 8.**
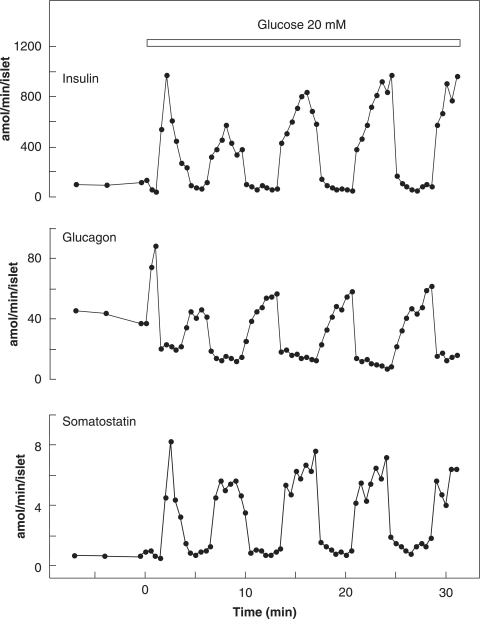
Effects of raising glucose from 3 to 20 mM on the release of insulin, glucagon, and somatostatin from a batch of 15 human islets. The hormones were measured in 30-second samples of the perifusate.

**Figure 9.**
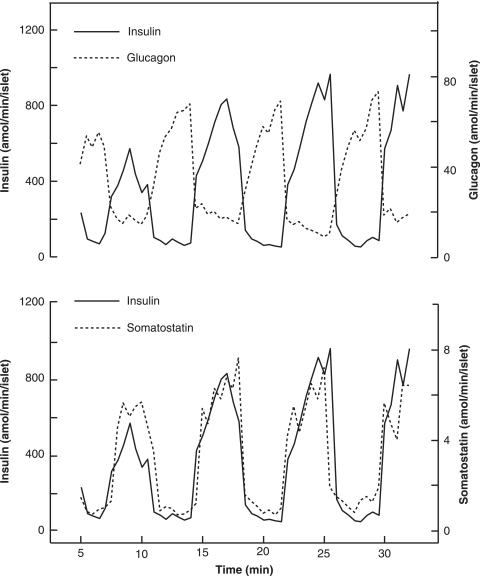
Relation between the repetitive release pulses of insulin and glucagon in the experiment shown in Figure 8. The insulin pulses are anti-synchronous to the glucagon pulses (upper panel) and coincide with the somatostatin pulses (lower panel).

**Figure 10.**
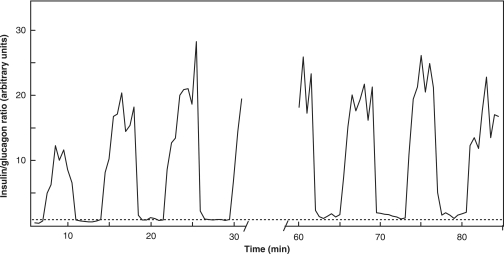
Variations of the insulin/glucagon ratio during superfusion of 15 human islets with 20 mM glucose. Sampling was interrupted for 28.5 min in the middle of the experiment. The insulin/glucagon ratio is given in arbitrary units with the average of nadir values set to 1.0 (dotted line).

Species differences in the cytoarchitecture of the islets make it important to analyse whether islet hormone pulses in rodents have their counterparts in man. It has been reported that [Ca^2+^]_i_ oscillations are poorly co-ordinated in β-cells (64) and totally asynchronous in α-cells (65) within human islets. Moreover, uncertainties exist whether the insulin oscillations in man are related to periodic variations of other islet hormones (66–69). Our recent studies of isolated human islets indicate that an increase of glucose from 3 to 20 mM induces release pulses of insulin, glucagon, and somatostatin at regular intervals (70). A representative experiment is shown in Figure 8. The relation between the hormones was similar as found during perfusion of rodent pancreas (see above). Accordingly, the pulses of insulin were anti-synchronous to those of glucagon (Figure 9, upper panel) but coincided with the somatostatin pulses (Figure 9, lower panel). Due to the anti-synchrony there were > 20-fold variations of the insulin/glucagon ratio during a pulse cycle (Figure 10). The glucose-induced generation of pulses resulted in marked increase of time-averaged release of insulin and somatostatin. In the case of glucagon the nadirs between the pulses were lower than at 3 mM glucose, resulting in a slight but significant suppression of average release.

## Clinical aspects on insulin and glucagon pulsatility

Pulsatile release of insulin into the portal vein generates 3–4-min oscillations, the amplitude of which is markedly suppressed after passage through the liver. The effects of insulin and other islet hormones are critically dependent on their possibilities to rapidly reach the target cells via fenestrations of the capillary endothelium. Besides the pancreatic islet cells also the liver cells are exposed to pronounced oscillations of insulin and glucagon. It is open for discussion to what extent target cells at other locations are directly affected by the periodic variations of the insulin/glucagon ratio.

A major advantage of periodic compared with continuous exposure to the hormones is to prevent down-regulation of their receptors. Several studies indicate that the cyclic variations of insulin and glucagon keep the receptors on the liver cells up-regulated (71–75). Pulse administration may be useful also in other kinds of receptor-mediated diabetes therapy. Attempts should be made to increase the number of glucagon-like peptide-1 (GLP-1) receptors, which are down-regulated in β-cells during hyperglycaemia and overt diabetes (76). Indeed, supraphysiological concentrations of GLP-1 have been found to correct both the deficient release of insulin and the excessive release of glucagon in type 2 diabetes (77). For practical reasons, pulse stimulation of the GLP-1 receptor should be performed with a stable analogue of GLP-1 (i.e. extendin-4).

Loss of regular oscillations of insulin is an early indicator of diabetes (12,13). The starting-point for treatment of diabetes is to investigate if the absence of regular insulin periodicity can be attributed to deficient β-cell rhythm or co-ordination. Efforts should be made to re-establish a normal periodicity. Interestingly, islets transplanted to the human liver have been found to release insulin in pulses (78). It is possible to generate regular insulin oscillations in the portal vein by bolus injections of the hormone into a hand or forearm vein. Sessions of pulsatile intravenous infusion therapy (PIVIT) have been reported to counteract renal and neural complications in diabetes (79–81). Recent progress in the understanding of islet hormone release urges for modifications of the PIVIT protocol to mimic the reversed pulses of insulin and glucagon.
